# Supplementary material for: Dynamic manipulation of droplets using mechanically tunable microtextured chemical gradients
Source: Nat Commun. 2021 May 25;12:3114. doi: 10.1038/s41467-021-23383-7 (PMC8149645; doi:10.1038/s41467-021-23383-7)
Supplement: Supplementary file 1 — Supplementary Information [file 41467_2021_23383_MOESM1_ESM.pdf]

Supporting Information

Dynamic manipulation of droplets using mechanically tunable microtextured chemical gradients

*Ali J. Mazaltarim,[1] John J. Bowen,[1] Jay M. Taylor,[1] Stephen A. Morin[1,2,3]**

[1]Department of Chemistry, University of Nebraska – Lincoln, Lincoln, NE 68588, USA
[2]Nebraska Center for Materials and Nanoscience, University of Nebraska – Lincoln, Lincoln, NE 68588, USA
[3]Nebraska Center for Integrated Biomolecular Communication, University of Nebraska-Lincoln, Lincoln, NE, 68588, USA
E-mail: smorin2@unl.edu

1. Microtexture characteristics

1.1. Calculating geometric features of surface wrinkles

The characteristic pitch (λ) of the wrinkles depends on the elastic moduli of the surface layer (E_f) and the bulk film (E_s) and the thickness of the surface layer (h_f)[1]:

$$\lambda = 2\pi h_f \left(\frac{\bar{E}_f}{3\bar{E}_s}\right)^{1/3} \quad \text{(S1)}$$

Here, $\bar{E}_x = \frac{E_x}{(1-v^2)}$ where E_x is the elastic modulus and v is Poisson's ratio for either the surface layer or the bulk film. We expected λ to remain relatively constant given the limited range of compressive strain used in our system. The critical strain where wrinkles begin to form is characterized by ε_{cs} and is also dependent on the elastic moduli[1]:

$$\varepsilon_{cs} = -\frac{1}{4}\left(\frac{3\bar{E}_s}{\bar{E}_f}\right)^{2/3} \quad \text{(S2)}$$

The amplitude of the wrinkles (A) is influenced by mechanical strain[1]:

$$A = h_f\left(\frac{\varepsilon}{\varepsilon_c} - 1\right)^{0.5} \quad \text{(S3)}$$

These dynamic wrinkles result surface roughness characterized by Wenzel's roughness factor (r) which is equal to the ratio of the actual area to the projected area[1].

$$r = \frac{actual\ area}{projected\ area} \quad \text{(S4)}$$

r can also be calculated using physically measured parameters assuming a sinusoidal surface microtopograph[1].

$$r = \frac{2\pi A}{\lambda R^2}\int_0^R \left(\frac{\lambda x^2}{4\pi^2 A^2} + x^2 sin^2\frac{2\pi x}{\lambda}\right)^{0.5} dx \quad \text{(S5)}$$

where R is the radius of the liquid microdroplet on the surface.

1.2. Estimate of the silica layer thickness (h_f)

Use of the above equations (equations (S1-S5)) require the thickness of the surface layer to be known. We estimated the thickness (h_f) of the silica layer that is formed by oxidation, as it is difficult to directly measure[2]. Briefly, we measured the critical strain ε_{cs} experimentally at 0.075, in order to quantify the moduli ratio ($\frac{3\bar{E}_s}{\bar{E}_f}$, equation (S2)), and calculated h_f (equation (S1)) using the experimentally measured λ of 61.8 ± 6.1 µm. We calculated h_f at 5.39 ± 0.5 µm which was in close agreement with previously reported values for h_f following a similar UVO procedure[2].

1.3. Calculating wrinkle amplitude (A) and roughness (r) and comparison with measurements

We used h_f and ε_{cs} to determine the theoretical wrinkle amplitudes (A) relative to ε_c and compared them with the experimentally measured amplitudes obtained via confocal microscopy (Fig. 1b). These results were in close agreement with one another. Further, we quantified the dynamic Wenzel roughness factor (r) at variable ε_c by directly measuring surface roughness using confocal microscopy (equation (S4), Fig. 1c). These results were also in close agreement with the theoretically calculated roughness based on the measured wrinkle characteristics (A and λ, equation (S5)).

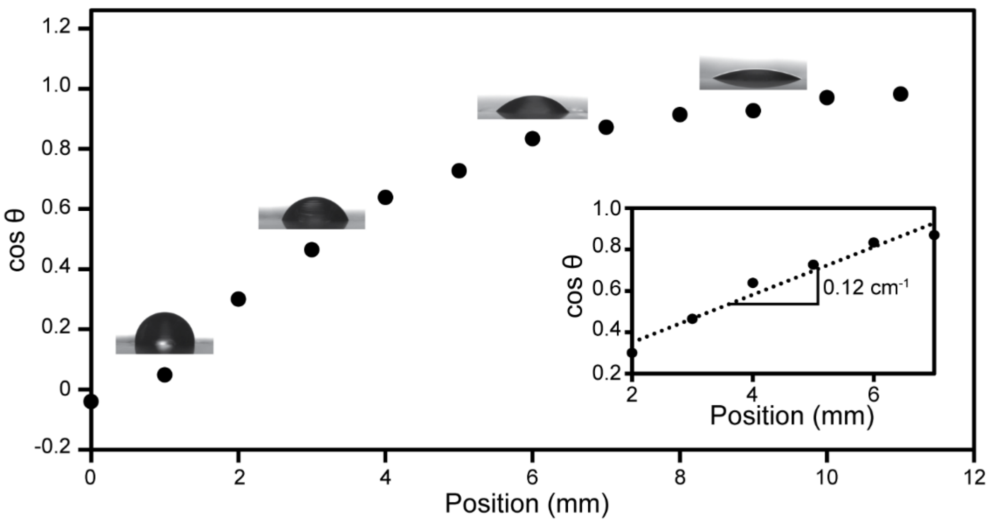

Supplementary Figure 1. Chemical gradient intensity at ε_c = 0.20. Variation in the measured contact angle (θ) as a function of position for a PDMS film at ε_c = 0.20. Inset: The contact angle variation for the linear portion of the gradient.

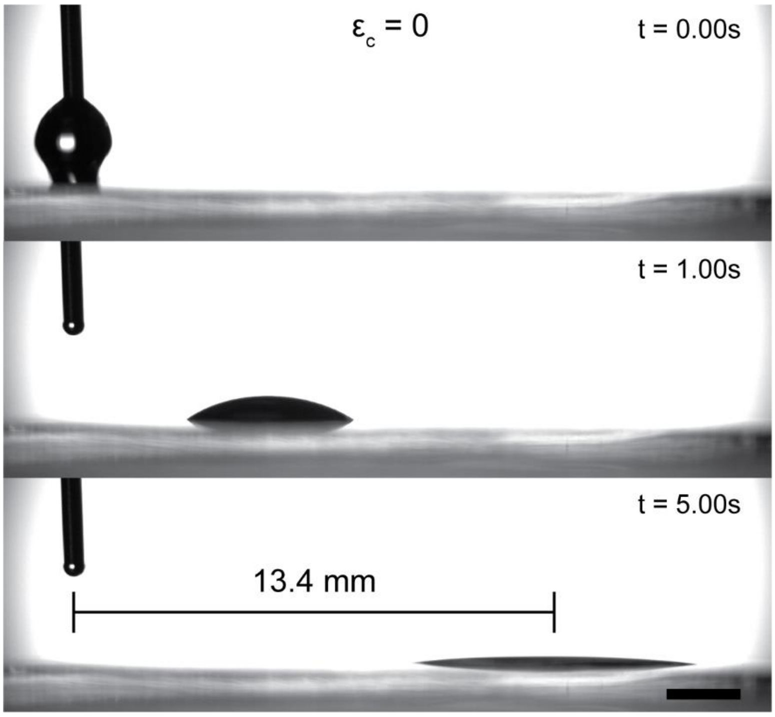

Supplementary Figure 2. Droplet transport on a strained film (ε_c = 0). Transport of a 3 µL DI water microdroplet deposited on a smooth gradient surface (r = 1). Time stamps are given as annotations. Scale bar = 2 mm.

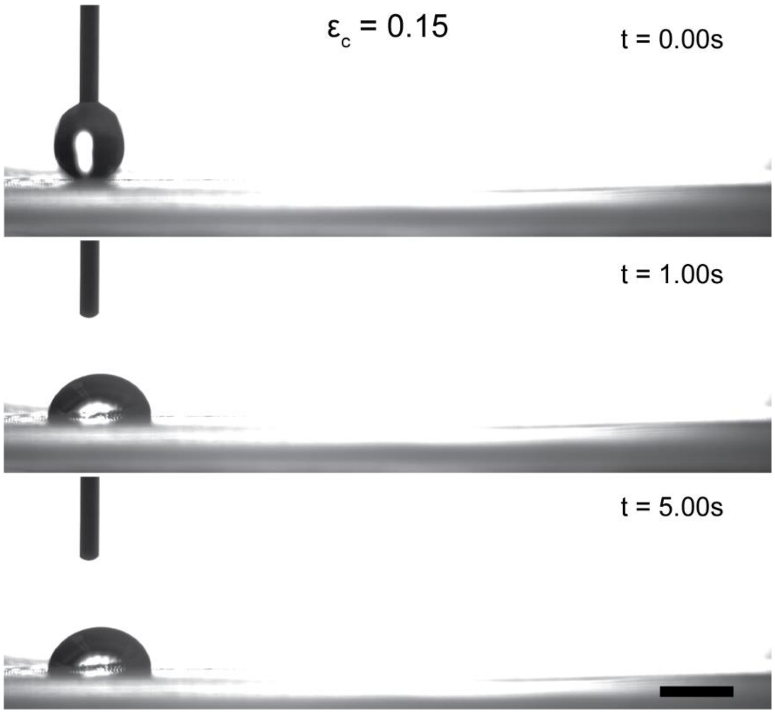

Supplementary Figure 3. Droplet transport on a compressed film (ε_c = 0.15). A 3 µL DI water microdroplet deposited on a rough gradient surface (r = 1.15 ± 0.03) did not move. Time stamps are given as annotations. Scale bar = 2 mm.

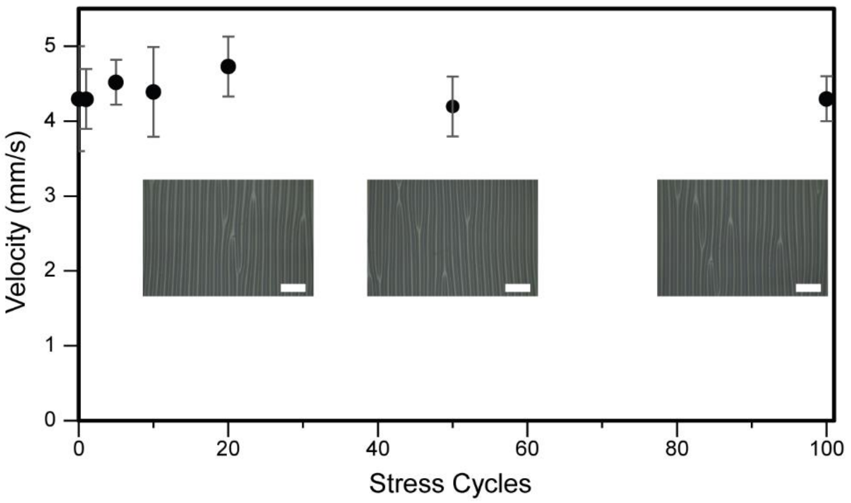

Supplementary Figure 4. Fluid transport performance upon successive stress cycles. Velocity measurement after successive stress cycles from $\varepsilon_c = 0$ to $\varepsilon_c = 0.2$. The droplet velocities were recorded using 3 μL DI H_2O droplets at $\varepsilon_c = 0$. Inset: Optical micrographs of the surface wrinkles ($\varepsilon_c = 0.2$) following stress cycling. Scale bars = 200 μm. (N = 5, data reported as $\bar{x} \pm s$ where $\bar{x}$ is the mean and s is the standard deviation).

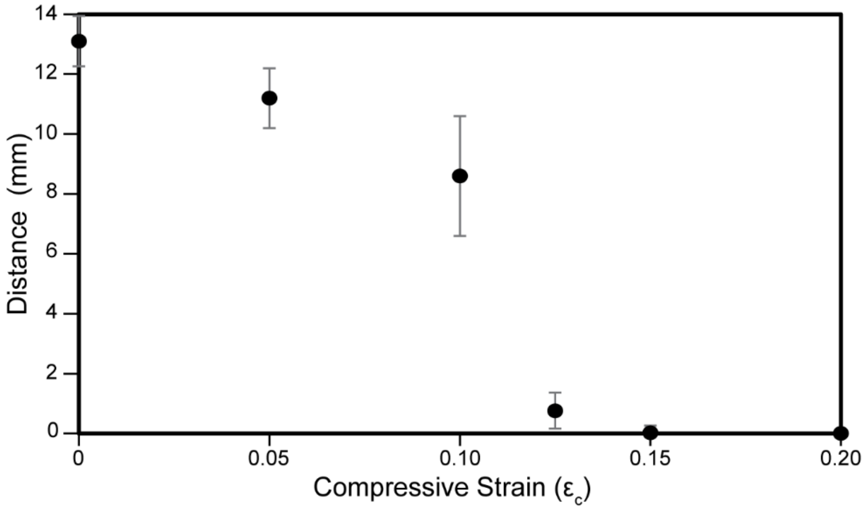

Supplementary Figure 5. Droplet displacement at various strain states. The total displacement distance that 3 µL DI H_2O microdroplets travel at differing compressive strain. (N = 5, data reported as $\bar{x}$ $\underline{+}$ s where $\bar{x}$ is the mean and s is the standard deviation).

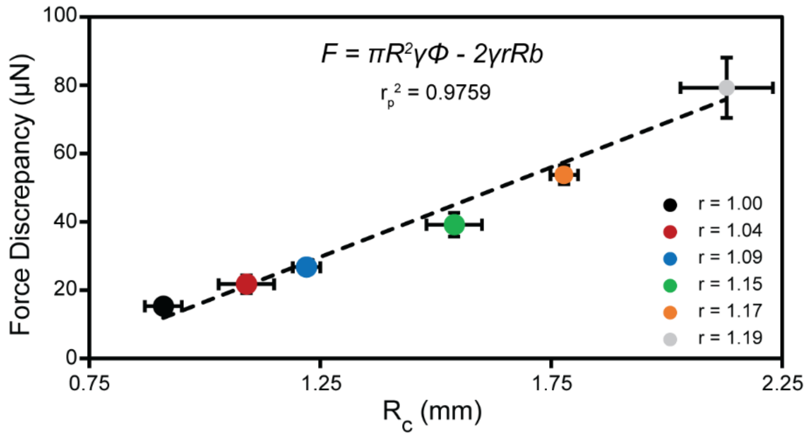

Supplementary Figure 6. Force difference relative to the critical radius at each respective strain state. A plot of the force discrepancy (equation (4)) using experimentally measured critical radii at varying ε_c. (N = 5, data reported as $\bar{x} \pm$ s where $\bar{x}$ is the mean and s is the standard deviation).

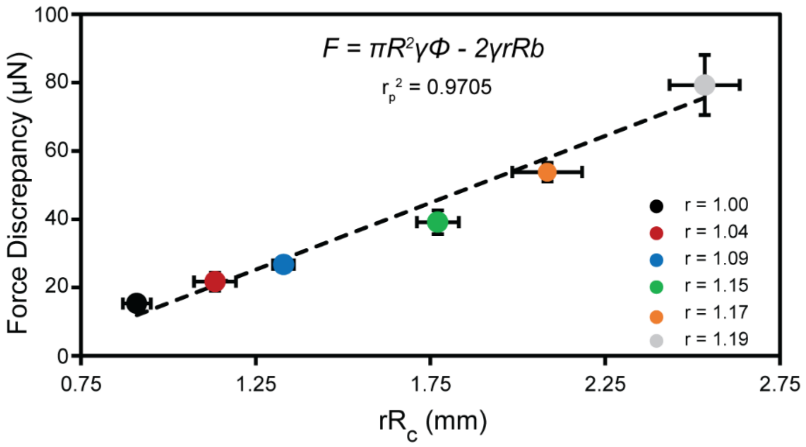

Supplementary Figure 7. Force difference relative to the critical radius multiplied by roughness (rR_c) at each respective strain state. A plot of the force discrepancy (equation (4)) using experimentally measured critical radii and roughness at varying ε_c. (N = 5, data reported as $\bar{x} \pm s$ where $\bar{x}$ is the mean and s is the standard deviation).

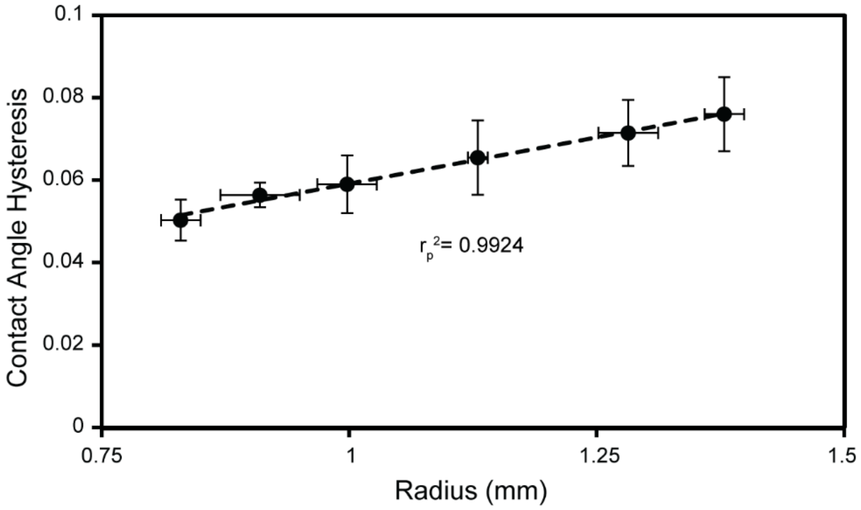

Supplementary Figure 8. Contact angle hysteresis (b). The measured contact angle hysteresis with respect to droplet radius as measured on a smooth gradient surface (r = 1). (N = 5, data reported as $\bar{x}$ $\pm$ s where $\bar{x}$ is the mean and s is the standard deviation). (N = 5, data reported as $\bar{x}$ $\pm$ s where $\bar{x}$ is the mean and s is the standard deviation).

Supplementary Movie 1.
Droplet transport on microtextured chemical gradient ($\varepsilon_c = 0$).

Supplementary Movie 2.
Droplet transport inhibition on microtextured chemical gradient ($\varepsilon_c = 0.15$).

Supplementary Movie 3.
Switching droplet transport "on" from an "off" state.

Supplementary Movie 4.
Toggling droplet transport between "on" and "off" states.

Supplementary Movie 5.
Toggling droplet transport on an inclined plane. Playback speed is half real time speed.

Supplementary Movie 6.
Toggling droplet transport on a declined plane. Playback speed is half real time speed.

Supplementary Movie 7.
Mechano-switchable self-cleaning surfaces.

Supplementary Movie 8.
Droplet sorting device. Playback speed is twice the real time speed.

References

1. Chung, J. Y., Youngblood, J. P. & Stafford, C. M. Anisotropic wetting on tunable micro-wrinkled surfaces. *Soft Matter* **3**, 1163–1169 (2007).
2. Glatz., B. A. et. Al. Hierarchical line-defect patterns in wrinkled surfaces. *Soft Matter* **11**, 3332-3339, (2015).
