# Supplementary material for: Dynamic manipulation of droplets using mechanically tunable microtextured chemical gradients
Source: Nat Commun. 2021 May 25;12:3114. doi: 10.1038/s41467-021-23383-7 (PMC8149645; doi:10.1038/s41467-021-23383-7)
Supplement: Supplementary file 3 — Description of Additional Supplementary Files [file 41467_2021_23383_MOESM3_ESM.pdf]

Description of Additional Supplementary Files

File Name: Supplementary Movie 1
Description: Droplet transport on microtextured chemical gradient ($\varepsilon_c = 0$).

File Name: Supplementary Movie 2
Description: Droplet transport inhibition on microtextured chemical gradient ($\varepsilon_c = 0.15$).

File Name: Supplementary Movie 3
Description: Switching droplet transport “on” from an “off” state.

File Name: Supplementary Movie 4
Description: Toggling droplet transport between “on” and “off” states.

File Name: Supplementary Movie 5
Description: Toggling droplet transport on an inclined plane. Playback speed is half real time speed.

File Name: Supplementary Movie 6
Description: Toggling droplet transport on a declined plane. Playback speed is half real time speed

File Name: Supplementary Movie 7
Description: Mechano-switchable self-cleaning surfaces.

File Name: Supplementary Movie 8
Description: Droplet sorting device. Playback speed is twice the real time speed.
